# Analyzing rescaling, discretization, and linearization in RNNs for neural system modeling

**DOI:** 10.3389/fncom.2026.1760701

**Published:** 2026-08-19

**Authors:** Mariano Caruso, Cecilia Jarne

**Affiliations:** 1 Escuela Superior de Ingeniería y Tecnología (ESIT), Universidad Internacional de La Rioja (UNIR), La Rioja, Spain; 2 Fundación I+D Software Libre (FIDESOL), Granada, Spain; 3 Departamento de Ciencia y Tecnología, Universidad Nacional de Quilmes (UNQ), Bernal, Buenos Aires, Argentina; 4 Consejo Nacional de Investigaciones Científicas y Técnicas (CONICET), Buenos Aires, Argentina; 5 Center of Functionally Integrative Neuroscience (CFIN), Department of Clinical Medicine, Aarhus University, Aarhus, Denmark; 6 Latin American Brain Health Institute (BrainLat), Universidad Adolfo Ibàñez, Santiago, Chile

**Keywords:** computational neuroscience, discretization, linearization, rescaling, RNNs

## Abstract

Recurrent Neural Networks (RNNs) are widely used to model neural activity in Computational Neuroscience. Here, we explore the mathematical foundations of three fundamental procedures that can be implemented: temporal rescaling, discretization, and linearization. These techniques provide crucial tools for characterizing the behavior of RNNs, offering insights into their temporal dynamics, facilitating practical computational implementation, and allowing for linear approximations for analysis. We discuss the flexible order in which these procedures can be applied, emphasizing their importance in modeling and analyzing RNNs for neuroscience and formally prove that these three operations commute pairwise. We also explicitly describe the conditions under which these procedures can be considered interchangeable. Our findings directly inform the design of biologically plausible *RNN* models for simulating neural dynamics observed in decision-making circuits and motor control, where temporal scaling and stability are critical for matching experimental recordings. Furthermore, we show that this exact commutativity guarantees the structural preservation of the network's controllability, preventing the emergence of inaccessible state-spaces under numerical discretization or temporal rescaling.

## 1 Introduction

**Figure 1:**
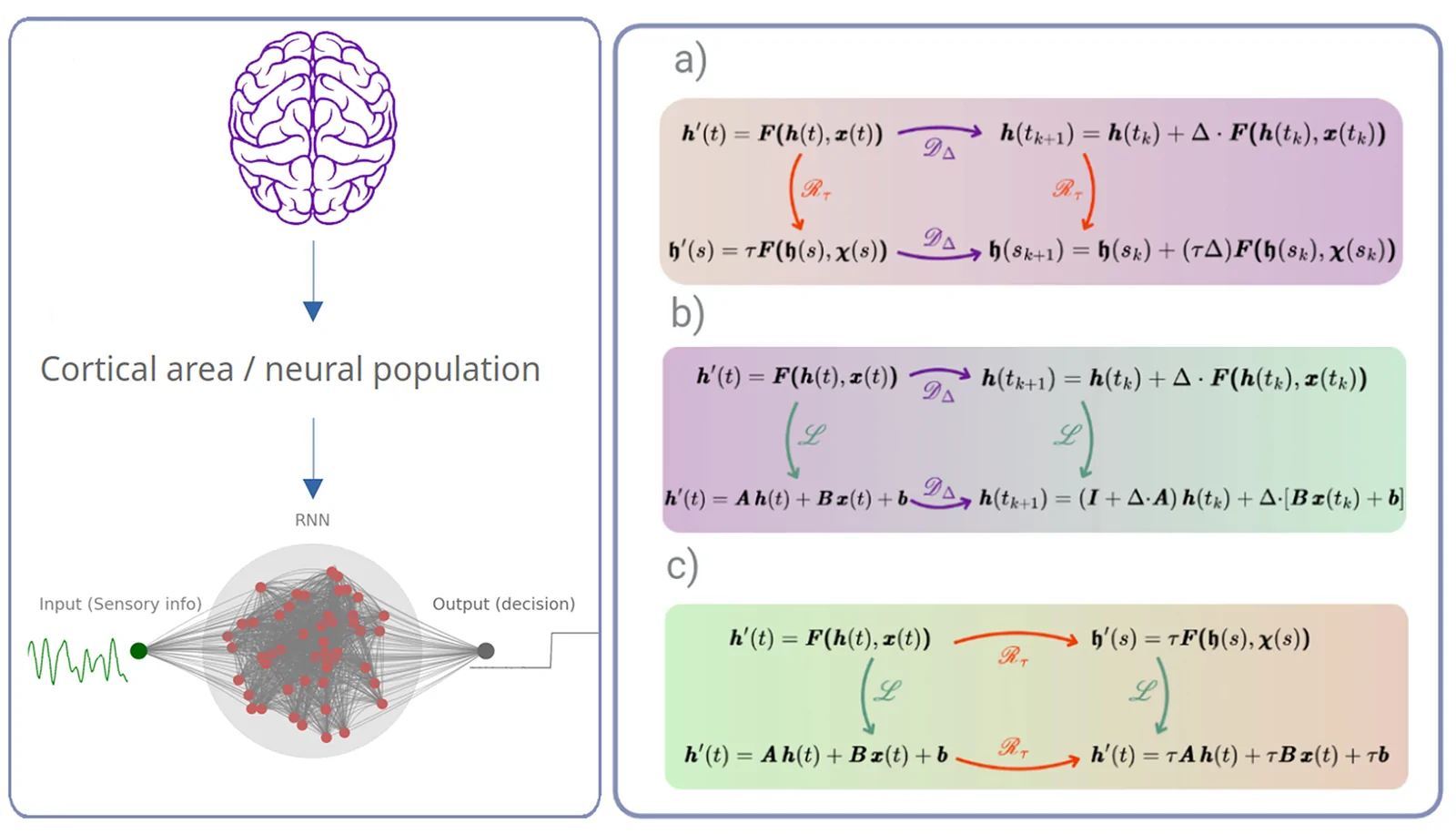
**Left panel**: Schema of how an *RNN* represents a cortical area or neural population. Operations over the *RNN*. **(a)** Scheme of how the equations are modified via temporal rescaling and discretization. **(b)** linearization and discretization process implemented in the equation for the activity. **(c)** Linearization and rescaling procedures implemented on the activity of units. In all panels, horizontal arrows ( → ) represent the application of the labeled operator to the equation at the origin of the arrow, yielding the expression at the arrowhead. Vertical arrows (↓) indicate the complementary operation applied to the alternative pathway, illustrating commutativity. Equations shown in the upper row of each panel correspond to the continuous-time formulation, while those in the lower row correspond to the transformed version. The result at each corner of the diagram is the same regardless of the order of application, confirming that the three operations commute pairwise, in the sense that for any pair among ℛ_τ_, 𝒟_Δ_, ℒ, their compositions are invariant under permutation.

*RNN*s are universal approximators of dynamical systems (Schäfer and Zimmermann, 2006; Funahashi and Nakamura, 1993). They have become invaluable tools in computational neuroscience over the last 40 years (Hopfield, 1984; Sussillo and Barak, 2013; Pandarinath et al., 2018). Networks typically represent certain cortical areas from an abstract set of recurrently connected units (see left panel of Figure 1). They are particularly effective in capturing the time-varying dynamics of neural processes, allowing us to simulate and analyse complex brain functions and interactions, such as motor control, decision-making and other complex processes (Russo et al., 2020; Bi and Zhou, 2020; Stine and Jazayeri, 2025; Jarne, 2021). Their ability to capture sequential dependencies and recurrent patterns makes them well-suited for tasks such as understanding information processing in the brain and decoding neural signals. *RNN*s also have various applications in machine learning, including sequential data analysis and time-series prediction (Zhang et al., 2023), and are also used to process brain data.

There are three main procedures we can consider applying to differential equations, particularly in the context of neuroscience and recurrent neural networks (*RNN*s). These procedures — temporal rescaling, temporal discretization, and linearization — are essential for characterizing the behavior of the systems represented by these models. We will discuss the outcomes of applying these procedures in different sequences and examine the conditions under which the order of application does not affect the results. In other words, we will explore when these procedures commute within the *RNN* framework. Additionally, we provide a brief introduction to each of these procedures. They are directly related to the design of biologically plausible *RNN* models used for simulating the neural dynamics observed in decision-making circuits (Wang, 2002) and motor control systems (Churchland et al., 2012), where temporal scaling and stability are critical for aligning with experimental recordings. While each of these operations is individually well understood, their pairwise commutativity within the *RNN* framework used in computational neuroscience has not, to our knowledge, been formally established in the literature. The present work fills this gap by providing explicit proofs and a unified diagrammatic representation.

Temporal rescaling can improve the numerical stability of solving differential equations. By rescaling the time variable, one can potentially reduce the condition number of the underlying linear system, which can result in more accurate and stable numerical solutions, especially when using numerical integration methods. This approach is especially useful in scenarios such as simulating long-term dynamics or processes that occur over a wide range of timescales. Temporal rescaling allows us to efficiently capture the behavior of a system. By adjusting the time units, we can focus computational resources where they are most needed, avoiding unnecessary computations at very short or very long times. It can help identify dominant modes, equilibrium points, or oscillations in the system and gain a better understanding of the underlying dynamics.

Transitioning from a continuous-time *RNN* to one suitable for computer implementation involves discretization. In continuous-time *RNN*s, dynamics is modeled using differential equations, which describe how neuron activations change continuously over time. However, computers operate in discrete time, meaning that they process information in discrete time steps. This is well known, and we daily employ numerical methods to approximate continuous-time behavior in a discrete-time framework. Given a small time step, often denoted as Δ, to divide time into discrete intervals. Then, we use the Euler method, or more advanced techniques to iteratively update the neuron activations at each time step (Cheney and Kincaid, 2018). Although a variety of numerical schemes can be used to discretize continuous-time RNNs, the present analysis focuses on the forward Euler method. This choice is not merely computational but also structural. The exact commutativity results established in this work rely on the fact that Euler discretization evaluates the vector field only once per time step, yielding an affine update that preserves the algebraic independence between discretization, temporal rescaling, and local linearization. In contrast, higher-order schemes such as Runge–Kutta methods construct the update rule through multiple intermediate evaluations of the non-linear vector field. These nested non-linear compositions introduce higher-order interaction terms that generally prevent the resulting discrete map from being identical to the one obtained by first linearising the continuous system and subsequently discretising it. Consequently, the exact commutativity proven here should be understood as a property of the Euler framework and not as a generic feature of arbitrary numerical integrators.

From a practical perspective, this restriction is also consistent with common RNN implementations in computational neuroscience and machine learning. Higher-order integrators increase the number of function evaluations required at each update step and therefore substantially raise both training and simulation costs, particularly when gradients must be propagated through long recurrent trajectories. Similar trade-offs have been discussed in the Neural ODE literature, where increased solver accuracy is often accompanied by significantly higher computational demands (Chen et al., 2018). Furthermore, the Euler formulation preserves a direct correspondence between the continuous-time firing-rate equation and its discrete implementation, maintaining a transparent interpretation in terms of passive decay and recurrent synaptic input. Investigating whether weaker forms of commutativity survive under higher-order or adaptive integration schemes remains an interesting direction for future work.

The key idea is to approximate continuous differential equations, such as those governing the *RNN*'s dynamics, using finite differences. This process effectively transforms continuous-time *RNN* equations into a discrete-time form. This transition allows us to use computers for training and inference while still capturing essential aspects of the continuous-time model's behavior, enabling the application of *RNN*s to develop computational models of the brain and cognitive tasks. It is important to note that despite this discretization, the behavior of the dynamical system can be characterized. The discrete-time *RNN*'s stability, for instance, can be assessed by examining the eigenvalues of its weight matrix, shedding light on the presence and stability of fixed points in the network dynamics (Sussillo and Barak, 2013; Jarne, 2023).

Linearization of dynamical systems simplifies complex systems into linear approximations, making it (sometimes) easier to analyse and understand their behavior. Linear systems are well-studied and have well-established mathematical tools for their analysis. Linearization helps to identify stable modes in cortical networks (Sussillo and Barak, 2013). Effects of different linearization mechanisms in *RNN*s have been discussed before (Pagan et al., 2023). The authors compare the *RNN* dynamics that can be written in terms of the “activations” or “activities,” meaning that *RNN*'s dynamics can be written in terms of the net inputs to each unit before the pointwise non-linearity or in terms of the output of each unit after the pointwise non-linearity.

We studied the interrelation of these three procedures: rescaling, discretization, and linearization, commonly used to create frameworks and explore *RNN*s and the performance, dynamics, and mechanisms when they are trained for different tasks.

The chosen assumptions and simplifications have direct consequences on the models and predictions. For example, in Wang et al. (2018), the authors considered a variant of the interval production task termed the cue-set-go (CSG) task and studied such in real data and trained *RNN*s. Neurons recorded during the CSG task display non-linear, non-monotonic activity that is temporally compressed on short interval trials and stretched on long interval trials, a phenomenon that has been termed temporal scaling. When all the neurons are temporally scaled by a certain factor without changing the response profile, the population activity goes through the same continuum of states, but only at a different speed. Therefore, in the state space, temporal scaling manifests as similar neural trajectories that evolve at different speeds. In that study, a linearization of the *RNN* dynamics was employed to investigate how temporal scaling arises from changes in input; the commutativity between rescaling and linearization formalized here provides a rigorous foundation for such approaches.

## 2 Methods

Consider a collection of *N* artificial neurons, each associated with a dynamic quantity termed activity, which is described by a function *h*_*i*_:[*a, b*] → ℋ⊆ℝ for *i* = 1, ⋯ , *N*. These *N* functions can be organized into a column vector $h={\left(h_{1},\cdots \;,h_{i},\cdots \;,h_{N}\right)}^{T}$, commonly referred to as the *hidden* layer of the *RNN*, where *T* indicates the transpose operation. The vector *h* encapsulates the network's activity state at time *t*, encompassing all *N* neurons. Additionally, there are *M* input functions, *x*_*k*_:[*a, b*] → *X*⊆ℝ for *k* = 1, ⋯ , *M*, which can be similarly arranged into a column vector $x={\left(x_{1},\cdots \;,x_{i},\cdots \;,x_{M}\right)}^{T}$. For recurrent neural networks, the activity vector *h* follows the differential equation:


$$\begin{array}{l} h^{\prime }\left(t\right)=-\lambda h\left(t\right)+\sigma \left(wh\left(t\right)+\tilde{w}x\left(t\right)\right). \end{array}$$ (1)

Here, *h*′(*t*) denotes the time derivative in the standard sense. The matrices *w* and $\tilde{w}$ have dimensions *N*×*N* and *N*×*M*, respectively, with the elements *w*_*ij*_ of *w* representing synaptic connections, similarly for $\tilde{w}$. The activation vector field σ maps ℝ^*N*^ to itself, and satisfies σ(0) = 0, reflecting the principle that neuronal activity cannot spontaneously regenerate; in other words, activating a neuron with zero initial activity yields zero output. Each component of σ is derived by applying a distinct non-linear function σ:ℝ → ℝ. For a vector φ∈ℝ^*N*^, expressed as φ = (φ_1_, ⋯ , φ_*N*_), the vector σ(φ) is given by (σ(φ_1_), ⋯ , σ(φ_*N*_)). Furthermore, λ^−1^ represents the decay time of each signal *h*_*i*_ when the network is entirely disconnected, meaning *w* = 0 and $\tilde{w}=0$.

The network's activity state is determined by Equation 1, which is updated as a result of the interaction between them via *w*, with external signals *x* influencing the neurons' activity according to $\tilde{w}$, along with some initial condition. Instead of starting from Equation 1, it may be useful to start from the “biased” version of the equation as follows


$$\begin{array}{l} h^{\prime }\left(t\right)=-\lambda h\left(t\right)+\sigma \left(wh\left(t\right)+b+\tilde{w}x\left(t\right)\right). \end{array}$$ (2)

In Equation 2, $b={\left(b_{1},\cdots \;,b_{i},\cdots \;,b_{N}\right)}^{T}$ is another column vector, and each component is the bias for each activity *h*_*i*_. We can write Equation 1 in terms of its components


$$h_{i^{\prime }}(t)=-\lambda h_{i}(t)+\sigma (\sum_{j=1}^{N}w_{ij}h_{j}(t)+b_{i}+\sum_{k=1}^{M}\;{\tilde{w}}_{ik}x_{k}(t)).$$ (3)

To better understand, we will work with a more compact notation:


$$\begin{array}{l} h^{\prime }\left(t\right)=F\left(h\left(t\right),x\left(t\right)\right). \end{array}$$ (4)

We use this compact notation $F\left(h\left(t\right),x\left(t\right)\right)=-\lambda h\left(t\right)+\sigma \left(wh\left(t\right)+b+\tilde{w}x\left(t\right)\right)$ and it will be useful to consider the matrices *A*: = *w*−λ*I* and $B:=\tilde{w}$, where *I* is the *N*×*N* identity matrix.

Although we don't see any “recurrence” relationship in the discrete mathematics sense, the germ of this concept is already present in Equation 4, indicating that changes in *h* over time depend on its current state.

As previously mentioned, we considered three main procedures that can be applied to this differential equation: temporal rescaling, temporal discretization, and linearization. We categorize the first two as different forms of rescaling, while we will address the discretization process separately. We will demonstrate that the outcomes of applying these procedures are independent of the order in which they are applied. In other words, we will prove that these procedures are mutually commutative under certain conditions.

### 2.1 Rescaling

Informally, time rescaling operator takes the activity function *h*(*t*) and *redraws* it on a new time scale. Instead of analyzing *h* as a function of time *t*, we define a new variable *s* such that *t* = τ*s*. If τ>1, the timeline is stretched (events occur later), and if 0 < τ < 1 the timeline is compressed (events occur more rapidly). Formally, the time rescaling function of parameter τ>0 is given by ϱ_τ_:*s*↦*t* = τ*s*. We introduce the rescaling operation by a factor τ for the activity *h*(*t*) as shown in Equation 5


$$R_{\tau }(h(t))(s)=(h{}^{\circ}\varrho_{\tau })(s).$$ (5)

We can apply the temporal rescaling operation (ℛ_τ_) on the network's activity vector 𝔥 = ℛ_τ_(*h*) given by the Equation 4. The external excitation vector χ = ℛ_τ_(*x*), and applying the chain rule for the time derivative *h*′, then 𝔥′(*s*) = τ*F*(𝔥(*s*), χ(*s*)). Finally, under rescaling operation ℛ_τ_, the expression Equation 2 takes the form of Equation 6


$$\begin{array}{l} {𝔥}^{\prime }\left(s\right)=-\tau \lambda 𝔥\left(s\right)+\tau \sigma \left(\omega \cdot 𝔥\left(s\right)+\tilde{\omega }\cdot \chi \left(s\right)+b\right). \end{array}$$ (6)

Comparing this expression with Equation 2, τ reappears in front of 𝔥 and σ. We can see how the temporal rescaling affects the characteristic relaxation time of the network in the absence of interaction λ^−1^↦(τλ)^−1^ and also the activation function. The maximum and minimum amplitude of the new activation function is modified by this operation. Every scaling transformation in the time domain has its counterpart in the frequency domain. Let *H*(ω) be the Fourier transform of *h*(*t*), then τ^−1^*H*(τ^−1^ω) will be the Fourier transform of ℛ_τ_(*h*(*t*)). This result can be compactly expressed in operational terms as $F•R_{\tau }=\tau^{-1}R_{\tau^{-1}}•F$, where ℱ denotes the Fourier transform, so that *H*(ω) = ℱ[*h*(*t*)](ω). It is noteworthy that a temporal rescaling in the model also affects the amplitude of the spectral activity.

### 2.2 Discretization

To compute Equation 4, in the sense of using a computer to perform simulations, it is always necessary to perform a discretization process. Let us consider the time interval to study such system, [*a, b*]⊂ℝ, we can apply a well-known procedure, which consists of cutting *n* pieces of equal size of Δ = (*b*−*a*)/*n*, to obtain a sequence {*t*_0_, *t*_1_, ⋯ , *t*_*n*_}, where *t*_0_ = *a*, and *t*_*n*_ = *b*. We introduce the discretization operation associated with the sequence of times *t*_*k*_ = *a*+*kΔ, k* = 0, ⋯ , *n*, for the activity *h*(*t*) as


$$D_{\Delta }(h(t))=h(t_{k}).$$ (7)

From the slicing {_*t*_*k*_}*k*_, the sequence of snapshots of corresponding neural activities {*h*(_*t*_*k*_)}*k*_ is defined. The operation Equation 7 can be applied also on the external excitation 𝒟_Δ_*x*(*t*) = *x*(*t*_*k*_). For very small Δ we obtain Equation 8


$$h^{\prime }(t_{k})≃\;[h(t_{k}+\Delta )-h(t_{k})]/\Delta ,$$ (8)

from the sequence of times, we have *t*_*k*+1_ = *t*_*k*_+Δ, valid for *k* = 0, ⋯ , *n*−1. By definition,


$$D_{\Delta }(h^{\prime }(t))=h^{\prime }(t_{k})$$ (9)

So, under Equation 9, now considered as an exact equality, the differential Equation 4 can be rewritten as *h*(*t*_*k*+1_) = *h*(*t*_*k*_)+*F*(*h*(*t*_*k*_), *x*(*t*_*k*_))Δ. Finally, applying the discretization operator 𝒟_Δ_ on the expression Equation 2 takes the form


$$\begin{array}{l} h\left(t_{k+1}\right)=h\left(t_{k}\right)-\lambda h\left(t_{k}\right)\cdot \Delta +\sigma \left(wh\left(t_{k}\right)+b+\tilde{w}x\left(t_{k}\right)\right)\cdot \Delta \end{array}$$ (10)

This serves as a recurrence relation; given the information of activity and excitation signals in a given slice, let's say at time *t*_*k*_, this relation gives us the value of the activity signal for the next slice at time *t*_*k*+1_. This form is well-suited for numerical computation, but its direct evaluation by hand is impractical owing to the repetitive nature of the iteration, particularly when the map *F* contains non-linear terms.

In Computational Neuroscience, the fixed points of the *RNN* models defined by Equations 1, Equation 2 are frequently used to simulate neural responses to static or slowly changing stimuli. Such equations are more commonly used explicitly in Computational Neuroscience, while Equation 10 is more common in machine learning, but they are closely related. Equation 10 has the same fixed point as Equation 1. Still, hyperbolic stability is obtained when eigenvalues have a magnitude less than 1. It should be noted that this eigenvalue condition strictly applies when the continuous-time system has already been linearised; for the full non-linear system, the correspondence between the magnitude of the discrete map's eigenvalues and asymptotic stability is only guaranteed locally, in a neighborhood of a fixed point. Hence, if a fixed point is stable for Equation 10, it is also stable for Equation 3, but the converse is not true (Zhu and Rosenbaum, 2023). In computational neuroscience, *RNN*s of the form of Equation 1 are studied using such mapping and also for their fixed point properties (Sussillo and Barak, 2013).

Additionally, the choice of Δ in the discretization can directly affect the model's ability to capture phenomena such as gamma oscillations (30–80 Hz) in neuronal activity, critical in attention processes (Buzsáki and Wang, 2012).

### 2.3 Linearization

Linear approximations around fixed points are widely used to study attractor dynamics in cortical networks (Brunel and Wang, 2001). Our framework formalizes conditions under which such approximations hold, which is critical for interpreting stability in models of persistent activity.

Since the non-linear object in *F* is exclusively in σ, linearization is a procedure related to the *activation field* and the type of signals (neuronal activity) we are interested in considering.

Within this activation field σ, let's now examine each function within σ:ℝ → ℝ and assume that σ(φ) is *k*-times differentiable at φ = 0. By Taylor's theorem, there exists a *remainder* function *R*_*k*_(φ) that allows us to write it as in Equation 11


$$\begin{array}{l} \sigma \left(\varphi \right)=\sigma \left(0\right)+\sigma^{\prime }\left(0\right)\varphi +\cdots +\frac{\sigma^{\left(k\right)}\left(0\right)}{k!}\varphi^{k}+R_{k}\left(\varphi \right) \end{array}$$ (11)

where σ^(*k*)^ is the *k*−order derivative of σ.

Activation functions, denoted by σ, are often chosen such that their tangent at the point φ = 0 has a slope of one. This means that near the origin, the activation function behaves similarly to the identity function, remembering that σ(0) = 0. Consequently, for sufficiently small values of φ, the activation function can be approximated as σ(φ)≃φ. This approximation holds within a regime where neuronal activity is constrained. We will refer to this as the “regular regime”. It is important to clarify that the linear approximation is not exclusive to the “regular regime.” Rather, within this regime, the neuronal activity is so minimal that the linear approximation is formally justified. This approximation is also applicable in scenarios involving long time periods. The rationale for this can be derived from the differential equation, where it is reasonable to assume a certain level of *neuronal tranquility* over time. By neuronal tranquility, we mean that as time progresses, either the matrix *A* (which is diagonalizable) has all its eigenvalues with negative real parts (a condition known as asymptotic stability), or the neuronal activation function, which integrates the weighted signals of each neuron, stabilizes the system by flattening the overall dynamics. The linearization of the activation function is formally defined as:


$$\begin{array}{l} L:\sigma \left(\varphi \right)\mapsto \varphi . \end{array}$$ (12)

Applying the linearization given by Equation 12, the differential Equation 4 simplifies to the expression shown in Equation 13:


$$\begin{array}{l} h^{\prime }\left(t\right)=Ah\left(t\right)+Bx\left(t\right)+b. \end{array}$$ (13)

Based on defined operations: rescaling ℛ_τ_, discretization 𝒟_Δ_, linearization ℒ, we will study the result of applying a sequence of two operations to the dynamics regulated by Equation 2.

Consider two computers and a scale change ℛ_τ_ that stretches τ>1 (compresses is given by τ < 1) the temporal samples. The following result can be anticipated intuitively: suppose a scale change ℛ_τ_ is performed and then discretized through 𝒟_Δ_ for use with computer 1, obtaining the sequence of activities 𝔥_1_(*s*_*k*_). On the other hand, if discretized with width Δ for use with computer 2 and then subsequently samples are separated (grouped) by a quantity given by τ: *t*_*k*+1_−*t*_*k*_ = τ(*s*_*k*+1_−*s*_*k*_), 𝔥_2_(*s*_*k*_) would be obtained, and it should be verified that 𝔥_1_(*s*_*k*_) = 𝔥_2_(*s*_*k*_). In this way, these two procedures: ℛ_τ_→𝒟_Δ_ and 𝒟_Δ_→ℛ_τ_ lead to the same results and are computationally indistinguishable. We can summarize both processes discussed and the resulting modifications of Figure 1a.

Let us now consider the linearization and ydiscretization process implemented in the equation for the activity as is shown in Figure 1b. In this case, we can see how both procedures: 𝒟_Δ_→ℒ and ℒ→𝒟_Δ_ lead to the same results and are also computationally indistinguishable.

Finally, let's consider the linearization and rescaling procedures as shown in Figure 1c. Again, we can see that the procedures ℒ→ℛ_τ_ and ℛ_τ_→ℒ are equivalent in the mentioned sense.

### 2.4 Operational equivalence of the procedures

To rigorously analyse how temporal rescaling (ℛ_τ_), discretization (𝒟_Δ_), and linearization (ℒ) interact, we adopt an approach based entirely on the structural invariance of the resulting equations. Rather than formulating an abstract operator algebra over heterogeneous mathematical spaces or dealing with incompatible domains, we evaluate commutativity by directly comparing the functional form of the expressions produced by each sequence of operations.

Let *A* and *B* be two distinct operations chosen from the set {ℛ_τ_, 𝒟_Δ_, ℒ}. If we first apply operation *A* to the original continuous-time *RNN* (Equation 2) and subsequently apply operation *B*, we obtain a final system equation denoted as *E*_*AB*_. Conversely, if we reverse the order of application, executing first *B* and then *A*, we arrive at the final system equation denoted as *E*_*BA*_.

Under this framework, we define a pair of operations to be operationally equivalent if and only if the resulting equations are analytically identical term-by-term, which we denote as Equation 14:


$$\begin{array}{l} E_{AB}\sim E_{BA} \end{array}$$ (14)

Whenever this relation holds, we can formally state that the procedures *A* and *B* are *commutative*.

This notion of operational equivalence formalizes the diagrammatic commutativity illustrated in Figure 1. It implies that if we follow two different sequences of operations to reach a common destination in the algebraic diagram, the final functional forms and updating rules obtained via both paths coincide exactly. In the following subsections, we demonstrate analytically that this identity holds strictly for each pair of procedures, confirming that the specific order in which they are deployed does not alter the final mathematical model of the *RNN*.

We now formally establish that the three operations introduced above commute pairwise. Throughout, we work with the general Equation 2.

#### 2.4.1 ℛ_τ_ and 𝒟_Δ_ commute

Let *h*(*t*) satisfy Equation 2 and let τ>0. Applying ℛ_τ_ followed by 𝒟_Δ_ yields the same discrete sequence as applying 𝒟_τΔ_ directly to the original equation (equivalently, 𝒟_Δ_ followed by a re-indexing of step size τ).

(ℛ_τ_→𝒟_Δ_) Define the rescaled variable *s* via *t* = τ*s*, so that *h*(*t*) = *h*(τ*s*) =:𝔥(*s*). Under ℛ_τ_, Equation 2 becomes


$$\begin{array}{l} {𝔥}^{\prime }\left(s\right)=\tau F\left(𝔥\left(s\right),\chi \left(s\right)\right), \end{array}$$ (15)

where **χ**(*s*) = *x*(τ*s*). Applying 𝒟_Δ_ to Equation 15 with the forward-Euler approximation gives


$$\begin{array}{l} 𝔥\left(s_{k+1}\right)=𝔥\left(s_{k}\right)+\tau \Delta \cdot F\left(𝔥\left(s_{k}\right),\chi \left(s_{k}\right)\right). \end{array}$$ (16)

(𝒟_Δ_→ℛ_τ_) Conversely, applying 𝒟_Δ_ first to Equation 2 with step size Δ produces


$$\begin{array}{l} h\left(t_{k+1}\right)=h\left(t_{k}\right)+\Delta \cdot F\left(h\left(t_{k}\right),x\left(t_{k}\right)\right). \end{array}$$ (17)

Setting *t*_*k*_ = τ*s*_*k*_, i.e., Δ_*t*_ = τΔ_*s*_, and substituting *h*(*t*_*k*_) = 𝔥(*s*_*k*_), Equation 17 becomes identical to Equation 16. Hence ℛ_τ_ and 𝒟_Δ_ are commutative.

#### 2.4.2 𝒟_Δ_ and ℒ commute

Applying 𝒟_Δ_ to the linearised equation yields the same discrete recurrence as linearising the discretized equation.

(ℒ→𝒟_Δ_) Applying ℒ (i.e., σ(φ)↦φ) to Equation 2 gives the linear system


$$\begin{array}{l} h^{\prime }\left(t\right)=Ah\left(t\right)+Bx\left(t\right)+b,\;A:=w-\lambda I,\;B:=\tilde{w}. \end{array}$$ (18)

Applying 𝒟_Δ_ to Equation 18 yields


$$\begin{array}{l} h\left(t_{k+1}\right)=\left(I+\Delta \cdot A\right)h\left(t_{k}\right)+\Delta \cdot Bx\left(t_{k}\right)+\Delta \cdot b. \end{array}$$ (19)

(𝒟_Δ_→ℒ) Applying 𝒟_Δ_ to the non-linear Equation 2 first gives


$$\begin{array}{l} h\left(t_{k+1}\right)=h\left(t_{k}\right)+\Delta \left[-\lambda h\left(t_{k}\right)+\sigma \left(wh\left(t_{k}\right)+\tilde{w}x\left(t_{k}\right)+b\right)\right]. \end{array}$$ (20)

Applying ℒ (replacing σ(φ)↦φ) to Equation 20 yields


$$\begin{array}{l} h\left(t_{k+1}\right)=h\left(t_{k}\right)+\Delta \left[-\lambda h\left(t_{k}\right)+wh\left(t_{k}\right)+\tilde{w}x\left(t_{k}\right)+b\right]\\ \;=\left(I+\Delta \cdot A\right)h\left(t_{k}\right)+\Delta \cdot Bx\left(t_{k}\right)+\Delta \cdot b. \end{array}$$ (21)

Equations 19, 21 are identical, so 𝒟_Δ_ and ℒ are commutative.

This result formalizes and extends the observation of Pagan et al. (2023), who noted that linearising before versus after the pointwise non-linearity yields equivalent dynamics; here we prove this equivalence holds exactly within the forward-Euler discretization scheme, for the full biased continuous-time *RNN* (Equation 2).

#### 2.4.3 ℒ and ℛ_τ_ commute

Linearising the rescaled equation yields the same result as rescaling the linearised equation.

(ℛ_τ_→ℒ) Under ℛ_τ_, Equation 2 becomes (see Section 2.1)


$$\begin{array}{l} {𝔥}^{\prime }\left(s\right)=-\tau \lambda 𝔥\left(s\right)+\tau \sigma \left(w𝔥\left(s\right)+\tilde{w}\chi \left(s\right)+b\right). \end{array}$$ (22)

Applying ℒ to Equation 22 gives


$$\begin{array}{l} {𝔥}^{\prime }\left(s\right)=\tau A𝔥\left(s\right)+\tau B\chi \left(s\right)+\tau b. \end{array}$$ (23)

(ℒ→ℛ_τ_) Applying ℒ to Equation 2 first yields Equation 18. Applying ℛ_τ_ to Equation 18 (using the same chain-rule argument as in Section 2.1) gives


$$\begin{array}{l} {𝔥}^{\prime }\left(s\right)=\tau \left[A𝔥\left(s\right)+B\chi \left(s\right)+b\right]=\tau A𝔥\left(s\right)+\tau B\chi \left(s\right)+\tau b. \end{array}$$ (24)

Equations 23 and 24 are identical, so ℒ and ℛ_τ_ are commutative.

The three propositions above establish the full commutativity diagram displayed in Figure 1. Every path through the diagram yields the same equation, regardless of the order in which ℛ_τ_, 𝒟_Δ_, and ℒ are applied.

### 2.5 Numerical verification of the commutativity properties

To illustrate the analytical results of Section 2.4, we implemented a small continuous-time *RNN* of *N* = 6 units receiving a single smooth input *x*(*t*) (a Gaussian pulse). The non-linear activation was σ(·) = tanh(·), which satisfies σ(0) = 0, σ′(0) = 1, placing the network in the *regular regime* under which the linearization ℒ is justified. Using the Euler discretization 𝒟_Δ_ with step Δ = 0.02 and a temporal rescaling factor τ = 1.6, we constructed the discrete recurrences corresponding to the two possible orders of each operator pair: (ℛ_τ_, 𝒟_Δ_), (𝒟_Δ_, ℒ), and (ℒ, ℛ_τ_).

For the pairs (ℛ_τ_, 𝒟_Δ_) and (ℒ, ℛ_τ_), each ordering was implemented as a separate Euler integration with its own vector field and/or step-size/time-scale schedule, so that the agreement between the two orderings is a non-trivial numerical check. For the pair (𝒟_Δ_, ℒ), the two orderings were implemented via two independent code paths: ℒ→𝒟_Δ_ discretizes the already-linearised continuous ODE (Equation 18), whereas 𝒟_Δ_→ℒ first discretizes the full non-linear vector field with forward Euler and then applies the substitution σ(φ)↦φ to the argument of σ within the resulting discrete update expression, without ever evaluating the linear vector field Equation 18 directly. Both procedures reduce analytically to the same affine recurrence (Equations 19–Equation 21), and their numerical agreement confirms this identity rather than merely reproducing a single computation under two names.

**Figure 2:**
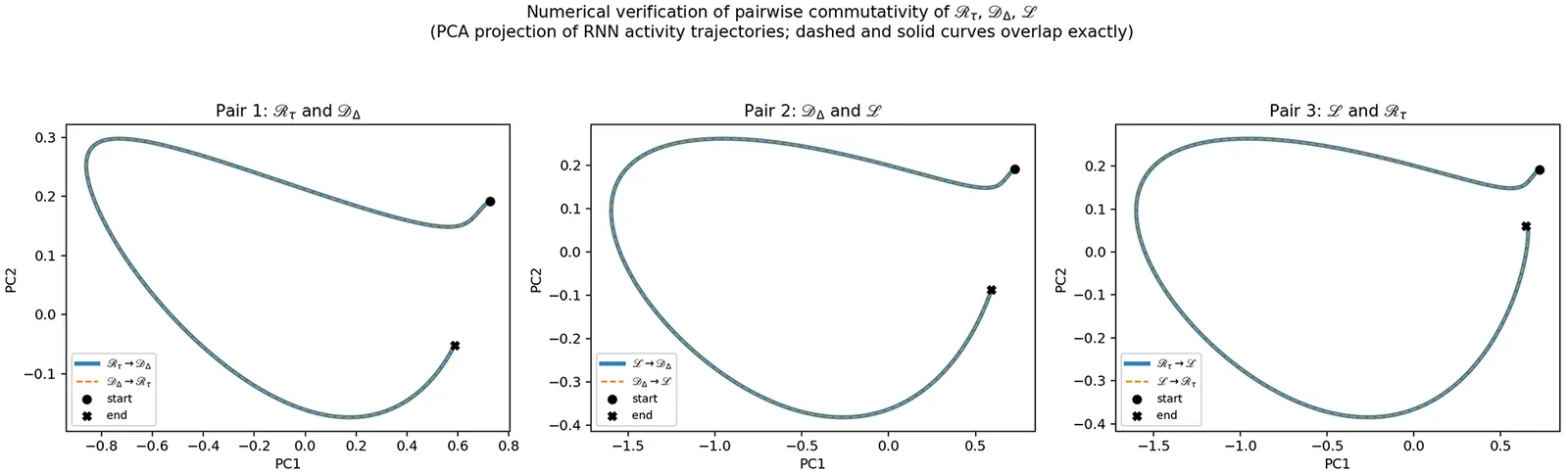
Numerical illustration of the pairwise commutativity of temporal rescaling (ℛ_τ_), discretization (𝒟_Δ_), and linearization (ℒ), for a small (*N* = 6) RNN of the form of Equation 2 driven by a smooth Gaussian-pulse stimulus *x*(*t*). Each panel shows the activity trajectory *h* projected onto the first two principal components (PC1, PC2) of the pooled activity, computed in the two possible orders of application of the corresponding pair of operators; the solid blue and dashed orange curves overlap exactly, with markers indicating the common initial (•) and final ( × ) states. **Panel a)** Pair (ℛ_τ_, 𝒟_Δ_): the trajectory obtained by rescaling time and then discretising (ℛ_τ_→𝒟_Δ_, Equation 16) coincides with that obtained by discretising the original equation with step τΔ and re-indexing the samples (𝒟_Δ_→ℛ_τ_, Equation 17); here the full non-linear vector field *F* is used in both orderings, with two genuinely different step-size/time-scale schedules. **Panel b)** Pair (𝒟_Δ_, ℒ): the trajectory obtained by linearising the continuous dynamics and then discretising (ℒ→𝒟_Δ_, Equation 19) coincides with that obtained by discretising the full non-linear equation and subsequently linearising the resulting discrete update expression (𝒟_Δ_→ℒ, Equation 21); the two trajectories are computed via two independent numerical procedures that both reduce analytically to the same affine recurrence *h*(*t*_*k*+1_) = (*I*+Δ*A*)*h*(*t*_*k*_)+Δ*Bx*(*t*_*k*_)+Δ*b*. **Panel c)** Pair (ℒ, ℛ_τ_): the trajectory obtained by rescaling and then linearising (ℛ_τ_→ℒ, Equation 23) coincides with that obtained by linearising and then rescaling (ℒ→ℛ_τ_, Equation 24); both reduce to 𝔥′(*s*) = τ*A𝔥*(*s*)+τ*B***χ**(*s*)+τ*b*. Note that the three panels correspond to different governing equations (non-linear in panel a, linearised in panels b–c, and additionally rescaled by τ in panel c), so the shapes of the trajectories differ *across* panels; the relevant comparison, which confirms the commutativity proofs of Section 2, is the exact overlap of the two curves *within* each panel. In all cases, the maximum absolute difference between the two orderings is at floating-point precision (~10^−16^, as reported by the accompanying script).

In every case, the trajectories obtained from the two orderings agreed up to floating-point precision (maximum absolute difference on the order of 10^−16^). Projecting the high-dimensional activity onto its first two principal components produced perfectly overlapping curves, confirming that the commutativity holds exactly under Euler discretization and providing a concrete numerical complement to the analytic proofs (see Figure 2). The code for reproducing these simulations is provided at https://github.com/katejarne/Analyzing_Rescaling_Discretization_Linearization_in_RNNs.

## 3 Discussion

We have delved into the fundamental mathematical framework of *RNN*s and introduced procedures such as temporal rescaling, temporal discretization, and linearization for characterizing the system's behavior. Each of these procedures is integral to understanding and modeling *RNN*s effectively.

Temporal rescaling is a crucial tool for studying *RNN*s over different time scales. Under a rescaling time operation, we can observe how the dynamics of the network change, potentially uncovering information about its behavior at different time resolutions. This procedure is particularly useful when dealing with networks that exhibit distinct behaviors or patterns over varying time intervals. This procedure is reversible, i.e., ℛ_τ_ has a unique inverse given by $R_{\tau^{-1}}$.

Discretization, on the other hand, plays an important role in implementing *RNN*s simulations on computers. It transforms the continuous-time differential equation into a discrete-time recurrence relation, making it computationally tractable. This is essential for simulating and analyzing *RNN*s in practice, allowing researchers to explore their behavior and capabilities systematically.

Linearization is another valuable tool that simplifies complex non-linear *RNN*s into linear approximations. This simplification aids in analyzing the network's behavior around specific operating points and designing control strategies. However, it is important to note that linearization is most effective when non-linearities are relatively small, and it may not be suitable for highly non-linear systems.

Discretization and linearization are both irreversible procedures. However, when both procedures are applied sequentially, they yield the same result regardless of the order in which they are applied.

These procedures discussed above are standard practices in the field of *RNN*s, and their order of application is often flexible, as they commute without altering the outcome. While they are powerful tools for modeling and analyzing *RNN*s, it's essential to choose the appropriate procedure based on the specific characteristics and goals of the neural network model being studied and compared with brain activity. For instance, temporal rescaling might distort the alignment between model dynamics and experimentally observed neural timescales (e.g., synaptic delays or oscillation frequencies). For example, gamma oscillations (30–80 Hz), which are tightly linked to attentional modulation and sensory processing (Buzsáki and Wang, 2012), require a discretization step Δ small enough to resolve sub-cycle dynamics; coarse time steps can alias these oscillations or suppress them entirely, leading to qualitatively incorrect network behavior. Similarly, synaptic delays on the order of a few milliseconds, which are physiologically critical in shaping the timing of cortical responses, may be inadvertently collapsed or distorted if that chosen Δ exceeds their duration. In such cases, the apparent dynamics of the discretized model may diverge substantially from those of the underlying continuous system, even when other qualitative features such as fixed points are preserved.

Similarly, discretization could introduce numerical artifacts that misrepresent continuous neural processes, such as spike timing precision. Furthermore, linearization risks oversimplifying non-linear phenomena critical to neural computation, like chaotic dynamics or bifurcations observed in cortical networks. Finally, it's worth considering that these procedures may not capture the full complexity of certain networks with strong, pervasive non-linearities or large state spaces, highlighting the need for a thoughtful approach to their application.

While the pairwise commutativity of temporal rescaling, discretization, and linearization is an elegant mathematical property, its primary value lies in resolving operational ambiguities in everyday computational neuroscience and machine learning workflows. We highlight three prominent practical scenarios: (1) A common paradigm in modern computational neuroscience involves “opening the black box” of trained RNNs to understand neural computation (Sussillo and Barak, 2013; Maheswaranathan et al., 2019). Because training via backpropagation through time (BPTT) requires discrete steps, researchers inherently obtain a discrete-time model, which they subsequently linearise to find fixed points and compute Jacobians. A persistent theoretical ambiguity is whether the stability properties (eigenvalues) derived from this discrete approximation faithfully represent the underlying continuous biological system. The exact commutativity between ℒ and 𝒟_Δ_ proven in this work acts as a formal guarantee: linearising the trained discrete model yields the identical dynamical landscape as linearising the original continuous ODE and subsequently discretising it. Consequently, researchers can confidently infer continuous biological attractors directly from discrete *in silico* Jacobians. (2) In studying cognitive processes such as motor timing (Wang et al., 2018), modelers often need to observe how a neural circuit behaves at different temporal speeds. If an RNN has already been trained with a specific integration step Δ and time constant τ, scaling the network's speed poses a methodological choice: should one scale the continuous equations and re-discretize (potentially requiring retraining), or simply manipulate the recurrent matrix of the already discrete model? The commutativity between 𝒟_Δ_ and ℛ_τ_ ensures that both approaches are analytically equivalent. Modelers can directly scale the discrete equations post-training without losing fidelity to the continuous system, thereby saving substantial computational resources and preventing numerical instability. (3) A standard procedure consists of characterizing the behavior of the system near its equilibrium points by computing the Jacobian matrix (linearization ℒ). When studying how these circuits adapt their processing speed to external demands through a temporal scale factor (rescaling ℛ_τ_), an ambiguity arises as to whether the local linear approximation is distorted or whether the nature of the attractors changes depending on the order of the analysis. The exact commutativity between ℛ_τ_ and ℒ provides a fundamental guarantee of invariance: linearising the temporally modified vector field produces exactly the same local phase portrait as temporally rescaling the original linearised system. The resulting eigenvalues are simply multiplied homogeneously by the factor τ, strictly preserving the signs, the directions of the eigenvectors, and the topological classification of the fixed point (whether it is a node, a focus, or a saddle point). This ensures that the computational and qualitative properties of the network do not suffer from geometric artifacts when altering the intrinsic speed of the system.

Beyond the neuroscience applications discussed here, the broader challenge of processing and protecting temporally and spatially structured medical data has motivated parallel advances in clinical deep learning. Recurrent and sequence-aware architectures are involved in multimodal medical imaging pipelines, including systems that must track patient identity across longitudinal studies while preserving clinically relevant features. Recent work has demonstrated that deep neural networks can simultaneously handle patient re-identification across diverse imaging modalities (Tian et al., 2025a) and protect sensitive identity information while retaining diagnostically critical signs in ophthalmic imagery (Tian et al., 2025b). These developments illustrate how the mathematical principles explored here – including stable temporal dynamics and appropriate discretization – are increasingly relevant not only for neural modeling but also for the design of trustworthy clinical AI systems that operate on sequential or multi-session medical data.

Crucially, the pairwise commutativity of these operations guarantees the structural invariance of the network's state-space reachability, also known as controllability (Sontag and Sussmann, 1997; Sontag, 1998). In realistic biological models where the input dimension is strictly less than the number of neurons (*M* ≤ *N*), the network achieves full state-space exploration not in a single computational step, but through temporal integration. By preserving the algebraic independence of ℛ_τ_, 𝒟_Δ_, and ℒ, the commutativity ensures that the system's controllability matrix maintains its full rank across domains. Consequently, if a continuous, non-linear *RNN* can theoretically be driven to an arbitrary target state via its external inputs, this topological property is faithfully preserved in its discretized, rescaled, and linearised computational counterpart (Sontag and Sussmann, 1997). This mathematical guarantee precludes the emergence of artificial “blind spots” or inaccessible sub-spaces in the simulated neural dynamics, regardless of the chosen integration step or temporal scale.

The commutativity results established here have direct practical implications for researchers building and training *RNN* models of neural dynamics. First, they justify the common but rarely formalized practice of interchanging the order of operations during model construction: for instance, a practitioner may choose to linearise an *RNN* analytically before implementing it in discrete time, or conversely, to implement the full non-linear system and linearise the resulting map, obtaining equivalent results in either case. Second, in training paradigms that involve temporal rescaling to match experimental recordings at different timescales [e.g., interval timing tasks as in Wang et al. (2018)], the commutativity with discretization guarantees that rescaling the continuous model before choosing a time step is equivalent to adjusting the step size after the fact, removing an otherwise ambiguous modeling decision. Third, commutativity with linearization ensures that fixed-point stability conclusions are consistent across the two common analytical pathways: whether one linearises the continuous system and then discretizes the resulting linear map, or discretizes the non-linear system first and then linearises the discrete map around a fixed point, the resulting recurrence matrix (*I*+Δ·*A*) is the same in both cases.

While these techniques offer powerful tools for understanding and modeling neural processes, future work could investigate whether analogous commutativity properties persist under higher-order numerical integrators, adaptive-step solvers, or linearizations performed around non-trivial attractors rather than near the origin.

## Data Availability

The original contributions presented in the study are included in the article/supplementary material, further inquiries can be directed to the corresponding author.
